# Immune and Metabolic Alterations in Children with Perinatal HIV Exposure

**DOI:** 10.3390/v15020279

**Published:** 2023-01-18

**Authors:** Louise D. V. du Toit, Andrea Prinsloo, Helen C. Steel, Ute Feucht, Roan Louw, Theresa M. Rossouw

**Affiliations:** 1 Department of Immunology, Faculty of Health Sciences, University of Pretoria, Pretoria 0001, South Africa; 2 UP Research Centre for Maternal, Fetal, Newborn and Child Health Care Strategies, University of Pretoria, Pretoria 0001, South Africa; 3 Maternal and Infant Health Care Strategies Research Unit, South African Medical Research Council, Pretoria 0001, South Africa; 4 Department of Hematology, Faculty of Health Sciences, University of Pretoria, Pretoria 0001, South Africa; 5 Department of Pediatrics, Faculty of Health Sciences, University of Pretoria, Pretoria 0001, South Africa; 6 Human Metabolomics, Faculty of Natural and Agricultural Sciences, North-West University, Potchefstroom 2520, South Africa

**Keywords:** neonates, infants, children, HIV-exposed, CHEU, mother-to-child transmission, innate immunity, adaptive immunity, metabolic changes

## Abstract

With the global rollout of mother-to-child prevention programs for women living with HIV, vertical transmission has been all but eliminated in many countries. However, the number of children who are exposed in utero to HIV and antiretroviral therapy (ART) is ever-increasing. These children who are HIV-exposed-but-uninfected (CHEU) are now well recognized as having persistent health disparities compared to children who are HIV-unexposed–and-uninfected (CHUU). Differences reported between these two groups include immune dysfunction and higher levels of inflammation, cognitive and metabolic abnormalities, as well as increased morbidity and mortality in CHEU. The reasons for these disparities remain largely unknown. The present review focuses on a proposed link between immunometabolic aberrations and clinical pathologies observed in the rapidly expanding CHEU population. By drawing attention, firstly, to the significance of the immune and metabolic alterations observed in these children, and secondly, the impact of their healthcare requirements, particularly in low- and middle-income countries, this review aims to sensitize healthcare workers and policymakers about the long-term risks of in utero exposure to HIV and ART.

## 1. Introduction

The widespread use of antiretroviral therapy (ART) and the ongoing commitment to end the human immunodeficiency virus (HIV) pandemic have seen a 31% decrease in new HIV infections and a 47% reduction in HIV-associated mortality across the globe in the past decade (2010–2020) [1]. New pediatric HIV infections have decreased by 53% over the same period and some countries have successfully eliminated vertical transmission through the introduction of comprehensive vertical transmission prevention programs [2]. This has been made possible with triple-combination ART (cART) for pregnant women, often started pre-conception, as well as post-exposure ART prophylaxis in newborns for a minimum of 6 weeks and longer for those considered to be at high risk or being breastfed.

Early infant diagnosis programs focus mainly on proving/disproving the HIV-negative status of the children and, until recently, children who were HIV-exposed-but-uninfected (CHEU) were seen as the successful outcome of the vertical transmission prevention programs. Only once ART had been established as the standard of care, coinciding with significant improvement in morbidity and mortality outcomes of children living with HIV, did CHEU become apparent as a risk group in their own right, with proven and persistent health disparities when compared to children who are HIV-unexposed-and-uninfected (CHUU) [3]. This has major implications for healthcare systems, especially for regions with a high maternal HIV prevalence, such as southern Africa where >20% of children are born to mothers living with HIV [4].

Immune dysfunction, severe infections, growth delays, cognitive and metabolic abnormalities, as well as increased morbidity and mortality have been reported in CHEU when compared to CHUU for reasons that remain largely unexplained [5,6,7,8,9]. It is still unclear whether maternal HIV infection and/or potential ART toxicity shape infant development, especially the maturation and function of the immune system, setting infants on a path towards suboptimal growth and development, as well as lifelong compromised immune function.

In the last few years, there has been increasing awareness not only of the complexity, but also the communal factors that drive the infectious morbidity, growth, and developmental delays, as well as the possible long-term metabolic sequelae in CHEU [9]. Furthermore, there is a better understanding of the intricate relationship between the immune and metabolic systems. Metabolites play critical roles in various steps of the immune response, such as macromolecule synthesis, intracellular signaling, post-translational modifications, and cell survival [10]. This review therefore explores the immunological and metabolic alterations that have been described in CHEU with the aim of elucidating the possible links between these immunometabolic aberrations and the clinical pathologies observed in CHEU.

## 2. The global Picture of CHEU: Prevalence, Morbidity, and Mortality

An estimated 15.4 million children globally are now living within the context of early-life exposure to HIV and/or ART [4]. This is 600,000 more children than the 2018 estimate of 14.8 million, with the great majority (13.2 million) of CHEU living in sub-Saharan Africa [11]. The geographic focus in terms of CHEU can further be narrowed to five high-burden countries, namely South Africa (3.5 million), Uganda (1.1 million), Mozambique (1.0 million), Tanzania (910,000) and Nigeria (880,000) [11]. From a public health perspective, it is additionally important to note that the prevalence of CHEU exceeds 15% of the general child population in five southern African countries, namely eSwatini (32.4%), Botswana (27.4%), South Africa (21.6%), Lesotho (21.1%) and Namibia (16.4%) [11].

The mortality of CHEU has been reported to be up to three times higher when compared to CHUU [12]. While the causes are multifactorial, infectious diseases are an important factor: CHEU have been reported to present two to three times more frequently with severe infections than CHUU in both the developed and developing world [13,14,15]. This is especially true in the first year of life, with gastrointestinal and lower respiratory tract infections commonly reported [16,17,18,19,20,21]. These severe infections lead to increased morbidity and mortality in these children, peaking at between two and six months of age [5,11,22]. Risk factors linked to this increased incidence of infectious diseases include high maternal HIV viral load (VL) and low CD4+ T-cell counts in blood [23,24]; vertical transmission of other infectious organisms [25]; malnutrition as well as lack of breastfeeding [20,26,27]; and sub-optimal maternal access or adherence to ART.

Apart from these factors, it has also been postulated that fetal exposure to HIV has the potential to leave an immune footprint due to the changes in the maternal immune milieu. Maternal immune activation can lead to placental inflammation, fetal innate immune activation, and priming of the adaptive immune responses, which have the potential to cause heightened inflammation on the one hand, but also modify children’s early life response to infections, on the other [28]. Importantly, CHEU have been found to have immunological abnormalities that may, in part, explain their increased susceptibility to pathogens [29], with altered innate and adaptive immune responses, described in more detail below.

Suboptimal growth and developmental outcomes in CHEU are another cause for concern. The growth and neurodevelopmental delay observed are potentially attributable to HIV-specific as well as general risk factors, which may be separate but are interlinked, ranging from exposure to HIV and ART, exposure to other pathogens notably *Mycobacterium tuberculosis* and cytomegalovirus, maternal depression, as well as socio-economic factors including lack of access to formal housing, safe water, and sanitation [30]. Disentangling these factors has proven to be challenging. For example, socio-economic and other social determinants of health are mediated through the known detrimental effects of suboptimal maternal and infant nutrition on childhood growth.

Antiretroviral therapy exposure in utero and post-partum is an important confounding factor in the multimorbidity described in CHEU. Pregnant women living with HIV are generally treated with two nucleoside reverse transcriptase inhibitors (NRTIs)—tenofovir disoproxil fumarate (TDF) and lamivudine—together with an integrase strand transfer inhibitor (INSTI), such as dolutegravir (DTG) or, at times, the non-NNRTI (NNRTI), efavirenz or a protease inhibitor (PI) [31]. While ART is generally safe and well-tolerated, TDF has known kidney toxicity and DTG has been associated with excessive weight gain, especially in women of African descent, which is often accompanied by metabolic changes, such as insulin resistance and hyperglycemia [32]. Due to limited ART choices in neonates, infants are generally given nevirapine, which is a first-generation NNRTI, with or without the NRTI, zidovudine (AZT). Both drug classes have potential toxicity in the neonate, especially mitochondrial and hematologic toxicity linked to AZT [33]. Research on optimizing maternal peri-conceptual and pregnancy ART regimens not only needs to focus on reducing vertical transmission, but also on minimizing the consequential, potentially detrimental, long-term health outcomes in the offspring [14].

The next section will briefly describe the immunometabolic aberrations described in CHEU and explore the possible underlying pathophysiology.

## 3. Altered Innate Immune Responses Due to Peripartum HIV and/or ART Exposure

The first line of defense against invading pathogens is the immediate, non-specific response of the innate immune system. In the case of CHEU, in utero exposure to ART and maternal HIV, together with the maternal cytokine environment, has been associated with chronic immune activation. Although it remains poorly understood, the resultant inflammation [5,34] has been linked to an altered cytokine profile that is postulated to support poor immune cell development and immune responses in the neonate [35]. Lohman-Payne et al. found increased prenatal interleukin (IL)-8 levels in CHEU due to exposure to maternal HIV and that this is indicative of prenatal immune activation [36].

Altered maternal gut microbiomes and mucosal dysregulation due to HIV infection with consequent microbial translocation may be a contributing factor to the immune activation observed in CHEU. Lipopolysaccharide (LPS), a major component of Gram-negative bacterial cell walls, is a marker of microbial translocation and has been associated with immune activation in individuals infected with HIV [37]. Increased levels of LPS have been reported to persist despite ART [35,38]. Lipopolysaccharide levels in the plasma of CHEU have been reported to be similar to those of CHUU and remain unchanged from birth until 6–12 years of age [39]. Notably though, the gut microbiome of breastfed CHEU has been reported to differ from those of CHUU [40]. These differences have been attributed to the changes observed in both the microbiome and breast milk oligosaccharide composition in mothers living with HIV [40]. The microorganisms present in breast milk act as probiotics that affect the composition of the intestinal microbiota of children. It has been reported that the gut microbiome of CHEU has significantly higher numbers of *Prevotella* and *Pseudomonas* species compared to that of CHUU [40]. *Prevotella* species have previously been proposed to induce activation of myeloid dendritic cells (mDCs) in individuals living with HIV, thereby driving mucosal immune activation and inflammation [41]. Due to the important role of the gut microbiome in the education of the infant immune system, further research should be conducted into other factors that could potentially affect the microbiome, such as the mode of delivery, gestational age at birth, maternal and infant diet, antibiotic therapy, genetic background of the host, geographic location, and differences in breastfeeding practices [42].

Exposure to antiretroviral agents has been associated with altered numbers of hematological parameters; including cells of the innate immune system (as reviewed by Abu-Raya) [43]. Children who are HEU have been found to have up to two-fold fewer neutrophils than their HUU counterparts, possibly as a consequence of in utero exposure to ART, and this reduced neutrophil count can persist up to 8 years of age [44]. Importantly, impaired neutrophil responses have also been noted in some CHEU [45,46,47]. Although a link between in utero ART-exposure and defective neutrophil oxidative burst has been observed [46] further studies are required in order to determine the effect of exposure to ART on impaired neutrophil function in CHEU. In addition, Musimbi et al. recently showed that genes associated with neutrophil-mediated immunity are repressed in CHEU [48]. The authors propose that this may be due to the trans-placental diffusion of soluble HIV proteins.

In addition, monocytes from CHEU have been shown to be activated to a greater extent, noted for up to 6 months of age, compared to those from CHUU [49]. This aberrant monocyte response leads to the expression of pro-inflammatory cytokines including tumor necrosis factor-alpha (TNF-α), interleukin (IL)-6, and IL-12 that may favor an antibacterial immune response over an antiviral response [45]. This is in contrast to what was observed in CHUU at 6 months of age, in which case monocytes responded more significantly to viral stimuli [45]. By one year of age, monocyte activation was, however, shown to be comparable in CHEU and CHUU [45].

The susceptibility of CHEU to viral infections may further be augmented by alterations in natural killer (NK) cells. Although to a lesser extent than in individuals living with HIV, NK cell counts in CHEU have been found to decline in the first year of life with cells showing increased differentiation and less killing activity, as demonstrated by lower expression of perforin and interferon-gamma (IFN-γ) [50,51].

Limited studies on antigen-presenting cells, specifically dendritic cells (DCs), have indicated that there is an increased responsiveness of mDCs in CHEU. Myeloid DCs of CHEU (up to 6 weeks of age) exhibit a heightened pro-inflammatory response towards pathogen-associated molecular pattern (PAMP) stimulation, particularly those associated with bacteria [50]. As the CHEU mature, this response to PAMPs becomes less evident, possibly resulting in an increase in susceptibility to bacterial pathogens [51].

Interestingly, there appears to be very few studies that have investigated platelets in CHEU. A study by Siawaya et al. found elevated numbers of platelets in CHEU compared to CHUU [52]. Notably, increased platelet activation in HIV-infected adults has been reported and levels of activation were found to persist despite cART [53]. Platelet-activating factor (PAF), produced by endothelial cells, macrophages, neutrophils, eosinophils, monocytes, mast cells, as well as platelets, is a potent mediator of inflammation and increased levels of PAF biosynthesis have been associated with systemic inflammation and immune activation [54,55]. This, in turn, has been suggested to contribute to an increased risk of HIV-associated neurocognitive disorders [56]. The role of platelets and PAF in HIV has been reviewed by Madzime et al. [57]. As such, investigation of platelets and, importantly, the biosynthesis of PAF, in the setting of CHEU could give insight into the neurocognitive abnormalities often observed in these infants.

The immune dysregulation observed in early infancy in CHEU is not restricted to the innate immune system and increased levels of pro-inflammatory markers at birth have been proposed to interfere with T-cell responses in the neonate [36], leading to an altered adaptive immune response that could increase the risk of morbidity and mortality in CHEU.

## 4. Altered Adaptive Immune Responses Due to Peripartum HIV and/or ART Exposure

The aberrant adaptive immune responses observed in CHEU may originate from alterations in T-cell ontogeny and differentiation [58]. Thymopoiesis, the process whereby T-cells mature and differentiate, takes place in the thymus. Thymic size is associated with thymic output as well as CD4+ T-cell counts [59]. Notably, albeit in a small study cohort (n = 20), CHEU have been found to have significantly smaller thymi than CHUU [60]. Altered thymic development and functionality observed in these infants culminate in lower mature CD4+ T-cell counts [61] and reduced T-cell receptor (TCR) diversity on naïve T-cells [62,63].

Clerici et al. described decreased CD4+ and CD8+ naïve T-cell percentages together with the presence of a significant number of HIV-specific CD4^+^ T-helper cells, and increased numbers of activated CD8+ T-cells. The latter, in conjunction with lower overall CD4+ T-cell counts, resulted in a lowered CD4/CD8 ratio, suggesting impaired immune development. In addition, the augmented memory T-cells together with diminished naïve T-cells suggest significant antigenic exposure in these new-borns. Finally, they observed an increase in double-negative T-cells, indicative of impaired thymic maturation in new-borns with in utero HIV exposure [61].

The persistence of these defects differs across studies. Kolte et al. reported that, while differences in thymic size were still evident at 15 months of age, no qualitative T-cell immune deficits could be detected [60]. In contrast, Clerici et al. found that impairments in CD4+ and CD8+ T lymphocytes persisted up to the pre-teen years and that the immunophenotype was, in fact, similar to those of children living with HIV [64]. These differences are difficult to explain, but could be due to the 11-year time difference between the studies. Pregnant women in the latter study were on AZT prophylaxis only, while women in the former study were on triple combination ART. Apart from potential drug toxicity, differences in restoration of CD4+ T-cell counts, and reduction of systemic immune activation and HIV viral load could have had an impact on T-cell populations in HIV-exposed-but-uninfected infants in Mozambique [24].

A study evaluating immunological changes in CHEU at birth, 12 months, and 6–12 years demonstrated increased expression of activation markers (CD38 and Human Leukocyte Antigen -DR isotype [HLA-DR]) on CD8+ T-cells from birth up to 12 months and increased numbers of CD38+ HLA-DR+ CD4+ and CD8+ T-cells at 12 months [39]. Increased programmed death receptor 1 (PD-1) expression on CD4+ T-cells observed at 12 months persisted up to 12 years of age while PD-1 expression on CD8+ T-cells was raised at 12 months but dropped over time [39]. Programmed death receptor-1 is a negative costimulatory receptor involved in the suppression of immune responses, and its expression is induced on activated immune cells, especially T- and B-cells. These results confirm that the immune system of CHEU is in a state of chronic activation even long after exposure to the virus has ceased.

Based on the hypothesis that in utero exposure to HIV can shape the adaptive immune response of an unborn child, a study assessed the TCR repertoire in cord blood samples of CHEU from the pre-ART era, using next-generation sequencing. The authors demonstrated less clonal diversity of the TCR beta chain of CHEU compared to those of CHUU from the same community. Reduced diversity is associated with a greater number of high abundance clonotypes and is indicative of clonal expansions. In this study, the expanded clonotypes were identified as HIV-1-specific in 6 out of 7 CHEU cord blood samples, suggesting that the clonal expansion was due to exposure to HIV antigen in utero [65].

Some chronic maternal viral infections, such as hepatitis B virus, can predispose naïve T-cells to be driven towards a pro-inflammatory T-helper (Th)_1_ response rather than the preferred immunoregulatory Th_2_ and T regulatory (Treg) responses usually observed in new-borns [66]. To investigate the effect of in utero HIV exposure on the naturally occurring Th profile and the predisposition of CD4^+^ T cells to acquire a Th_1_ phenotype in vitro, 50 HEU new-borns were enrolled in a study in New Mexico. The CHEU group had smaller subsets of Th_1_, Th_2_, Th_17_, and CD4+ CD25 + + (presumably Treg) T-cells compared to their unexposed counterparts. The reduced proportions of these Th cells found in CHEU resulted in impaired effector cytokine production [58].

Brito-Pérez et al. observed that the number of Th_0_ cells, defined as antigen-primed T-cells that do not gain an effector function despite stimulation from the TCR or locally associated cytokines, were elevated in CHEU. This would imply that the stimulation received by the T-cells is insufficient for them to differentiate into Th effector cells [58]. They postulate that this might be due to alterations in the in vivo effects that induce differentiation, mostly driven by the innate immune system. Indeed, previous studies have reported that CHEU have lower percentages of NK cells and that these cells have a reduced capacity to produce IFN-γ, a cytokine that plays a critical role in both innate and adaptive immunity [51]. In addition, the production of IL-12, a cytokine that is principally involved in the differentiation of naïve T cells into Th1 cells, has been shown to be reduced in the cord blood mononuclear cells of HEU new-borns [67]. It is therefore possible that abnormalities in IL-12 regulation may contribute to the decreased cellular responses observed in CHEU.

The effect of CHEUs’ reduced ability to produce effector Th cytokines is also evident in their deficient cell-mediated immune response towards vaccines [68,69]. For instance, diminished numbers of proliferating T-cells and reduced T-cell functionality were found in CHEU following vaccination with Bacillus Calmette-Guérin (BCG) and tetanus toxoid [68,70]. In addition, the increase in CD8+ regulatory T-cell counts observed in CHEU may lead to weaker T-cell responses against vaccines and, indeed, infections [70].

Recently, the differences in maternal microchimerism between CHEU and CHUU were investigated as a potential mediator of altered T-cell responses [71]. Microchimerism is the transfer of maternal cells to the fetus during pregnancy. Modest numbers of these maternal cells persist in the infant after birth. Initiation of ART during pregnancy was found to result in lower levels of microchimerism in CHEU compared to those observed where the mother had initiated ART before the pregnancy or in CHUU. Maternal microchimerism was associated with an improved polyfunctional CD4+ T-cell response to BCG. This implies that early ART initiation, leading to increased CD4+ T-cell counts, as well as the recovery of immune dysregulation or placental inflammation, may ‘normalize’ the extent of maternal microchimerism in CHEU. The authors suggested that lower maternal cells in CHEU at birth may, at least in part, be responsible for the attenuated response of CHEU towards the BCG vaccine [71].

Dysfunctional B-cells could also contribute to suboptimal responses to vaccines. While similar B-cell subsets have been found in CHEU and CHUU during the first 18 months of life [72], CHEU have been reported to have increased apoptosis in B-cells [64]. In addition, dysregulated B-cell homeostasis, as evidenced by reduced levels of tissue-like memory and immature-transitional B-cell subsets, have been described in CHEU up to 6 months of age [73,74,75]. Once again, it appears that the maternal HIV viral load greatly influences B-cell levels [39,72]. This impaired B-cell homeostasis and function may contribute to the increased susceptibility of CHEU to potentially life-threatening diseases [75].

A dysregulated immune environment, mostly characterized by a pro-inflammatory milieu, has also been described in CHEU. For instance, Kashara et al. reported increased plasma levels of the pro-inflammatory cytokines, IL-1β and TNF-α, in CHEU [76]. Furthermore, lower levels of the anti-inflammatory cytokine, IL-10, were observed in these infants. Significantly higher levels of TNF-α and IFN-γ were also found following polyclonal activation of T-cells isolated from these CHEU. Since this cytokine imbalance was not related to T-cell subset counts or HIV-1 antigen sensitization, the authors postulated that it resulted from ART exposure in utero [76]. Interestingly, a study by Falconer et al. also observed increased secretion of TNF-α and IFN-γ in CHEU following BCG vaccination compared to CHUU. However, this was only noted in those infants born to woman with evidence of latent *Mycobacterium tuberculosis* infection [77].

As shown by Kashara et al., in addition to the consequences of in utero exposure to HIV, the effect of ART exposure cannot be discounted. Perinatal exposure to ART has been shown to impair adaptive immune cells and responses. A South African study evaluated the effect of ART exposure on Th cell responses in CHEU. Both mothers and neonates received a short course of zidovudine-lamivudine. Weaker HIV-stimulated T-helper cell reactivity was observed in CHEU exposed to ART, suggesting that ART exposure might negatively influence HIV-stimulated T-cell responses [78]. Another study of CHEU exposed to zidovudine for a minimum of two weeks reported reduced absolute CD4+ T-cell counts at 24 months of age [79].

The underlying mechanisms causing adverse events in CHEU are complex and multifactorial requiring more harmonized collaborative clinical cohorts of adequate size to identify and link the mechanisms involved as well as the clinical impact thereof [80]. One area of research that is gaining rapid traction in HIV research is that of metabolomics. The effect of in utero HIV-exposure, as well as that of ART particles, on metabolic dysregulation in CHEU may give further insights into the immunological consequences of this exposure in CHEU.

## 5. Metabolomic Abnormalities of HIV-Exposed Uninfected Children

The association between immune cell function and cellular metabolism has been well established [81,82]. In this section, the metabolomic abnormalities in CHEU will be discussed.

Mitochondrial dysfunction, or mitochondrial toxicity, including mutations in mitochondrial DNA (mtDNA), alterations in mtDNA levels, and aberrant mitochondrial histological morphology, has been reported in children perinatally exposed to HIV and ART [83,84,85]. the primary role of the mitochondrion is oxidative phosphorylation for the generation of adenosine triphosphate, which is essential for numerous pathways of intermediary metabolism, such as amino acid and organic acid metabolism, and fatty acid oxidation, all of which could be affected by mitochondrial toxicity [86]. In addition to generating the bulk of the cellular energy, mitochondria also control redox homeostasis, Ca^2+^ signaling, iron metabolism, innate immunity, and apoptotic cell death [87]. Mitochondria function and are regulated by approximately 1500 proteins encoded by two genomes—the nuclear and mitochondrial genome [88]. mtDNA is maintained by polymerase gamma, but this enzyme is also a sensitive target for inhibition by nucleotide reverse transcriptase inhibitors, thus blocking DNA polymerization reactions [89]. Not only ART-treatment but also HIV-infection is known to lead to mitochondrial dysfunction [90].

Mitochondrial dysfunction further affects immune function, where fatty acid oxidation both modulates macrophage inflammatory function and regulates T-cell responses while the metabolism and availability of many amino- and organic acids play a vital role in the effective functioning of the immune system [91]. In CHEU, dysfunction of normal oxidative phosphorylation can lead to clinical manifestations such as hypoglycemia, hyperketonemia, acute liver injury, and myopathy [86].

Jao et al. evaluated 37 acylcarnitines and three branched-chain amino acids in 6-week-old CHEU exposed in utero to ART (zidovudine/lamivudine/nevirapine) and postnatal zidovudine or nevirapine, as indicators of changes in biochemical fuel metabolism and insulin sensitivity. They found a strong correlation between long-chain acylcarnitines and lower levels of mtDNA content as well as between short-chain and branched-chain amino acid-related acylcarnitines and higher levels of mtDNA content in CHEU exposed specifically to AZT [92]. Additionally, another study by Jao et al. found that in utero HIV/ART and postnatal AZT or NVP exposure in infants was associated with abnormal acylcarnitine and branched-chain amino acid profiles at 6 weeks of age, which are indicative of altered insulin metabolism, and may manifest later in life as a predisposition to insulin resistance [93]. Taken together, these studies point to altered fuel utilization brought on by mtDNA dysfunction, the impact of which may negatively affect neonatal growth and weight gain. Previously described aberrations such as growth impairment, cardiac abnormalities, and neurodevelopmental delays in CHEU [94] could all be attributed to, or aggravated by, mitochondrial dysfunction [95]. These changes need to be explored further, together with the long-term risk of metabolic complications such as diabetes or obesity [96].

In a Canadian cohort of CHEU exposed to cART (a combination of two NRTIs and either a NNRTI or a PI) in utero and to AZT at delivery or for 6 weeks postnatally, plasma lactate levels were found to be increased above the normal limit at least once during the first 6 months of life as compared to CHUU. The conversion of pyruvate to lactate is the most well-known metabolic perturbation in mitochondrial dysfunction [97], therefore it was not surprising that the researchers hypothesized that the hyperlactatemia was a consequence of persistent mitochondrial toxicity due to the infants’ exposure to AZT. Hyperlactatemia is an important clinical consideration since it can lead to metabolic acidosis if not detected and managed appropriately [98].

Abnormal acylcarnitine profiles in newborn CHEU exposed to AZT were also demonstrated in a study by Kirmse et al. [99]. This finding is potentially indicative of perinatal fatty acid oxidation dysfunction. It also appears that aberrant acylcarnitine profiles, and therefore abnormal fatty acid oxidation, are even more likely to occur when in utero exposure to ART is to a PI-containing regimen. There are many ways in which PIs might affect fatty acid oxidation. Firstly, PIs are known to negatively impact lipid metabolism and may suppress fatty acid oxidation directly by inhibiting CD36 (a fatty acid translocase) [100,101]. Evidence also suggests that PIs can cause mitochondrial dysfunction by triggering mitophagy pathways and through the generation of reactive oxygen species (ROS) [102,103]. PIs can also contribute to the development of insulin resistance, which in turn perturbs fatty acid oxidation, leading to abnormal acylcarnitine levels [104]. In summary, the authors note that the abnormal fatty acid oxidation observed with the use of PIs may be multifactorial and includes the direct suppression of fatty acid oxidation, insulin resistance through abnormal acylcarnitine levels, and mitochondrial dysfunction [99].

Two specific metabolites, hypoxanthine and 3-hydroxybutyric acid, have also been shown to differ between CHEU and CHUU at birth, with hypoxanthine levels increased and 3-hydroxybutyric acid levels decreased in CHEU vs. CHUU [105]. Both hypoxanthine and 3-hydroxybutyric acid are known markers of mitochondrial dysfunction [97]. Another study conducted at birth, with infants exposed in utero to AZT, showed that peroxidized lipids, which are products of ROS, were elevated in CHEU compared to CHUU. Elevated ROS is a hallmark of mitochondrial dysfunction and deemed a key driver in mitochondrial dysfunction pathology [106]. Lipid metabolism showed further dysregulation, demonstrating an increase in triglyceride species which translates to a decrease in phospholipids in CHEU vs. CHUU. Phospholipids are essential building blocks in the membranes of cells and organelles and a decrease in these lipids could indicate dysregulation of many biological processes including growth and immune function [107].

## 6. Implications for Infant Growth and Development

The investigation of CHEU outcomes has not only been challenging due to the various contributing factors, but also because of repeatedly changing interventions and ART guidelines. Also, the medium-to long-term health impacts can best be studied through longitudinal cohort studies, which are labor intensive, time-consuming, and limited by participants becoming lost to follow-up. In addition, growth, neurodevelopmental, and immunological outcomes are intricately linked, adding further complexity.

Multiple studies have shown that growth deficits in CHEU are already appreciable at birth [94]. Suboptimal fetal growth is then potentially compounded by postnatal growth deficits, which are greatly impacted by infant feeding, especially the quality and duration of breastfeeding. Childhood growth is also influenced by the overall health of the child, with increased infections, both in terms of number and severity, further limiting growth and development. Infectious risk is linked to the described immune abnormalities in CHEU, but also to suboptimal breastfeeding practices and poor socio-economic circumstances.

Unfortunately, our understanding of the clinical impact of peripartum HIV and ART exposure remains incomplete. Several studies have reported on clinical outcomes, such as increased susceptibility to infection in CHEU, however, these studies did not perform immunological analysis to explore the possible causes [108,109,110]. Conversely, many studies reporting on changes in immunity did not correlate these changes with clinical outcomes [64,72,111] leaving the clinical significance of these findings unclear [112]. Harmonized collaborative clinical cohorts of adequate size are crucial to investigate the underlying mechanisms causing adverse events in CHEU as well as the clinical impact thereof [80].

## 7. Conclusions

While the reasons for worse clinical outcomes amongst CHEU remain unclear, this review aimed to shed light on the interconnectedness of the immunological and metabolomic aberrations in CHEU that culminate in altered innate and adaptive immune responses, causing immune activation, on the one hand, and increased susceptibility to infection, on the other. Concurrently, metabolomic aberrations manifest as mitochondrial toxicity, altered insulin metabolism, hyperlactatemia, and abnormal amino acid profiles that lead to growth delays. It is essential that the long-term risks of in utero exposure to HIV and ART in CHEU be fully appreciated so that suitable mitigation strategies can be employed on an individual as well as public health basis.

## 8. Future Directions

More studies are required to clarify the link between immunometabolic alterations and clinical outcomes in CHEU. In addition, the growth and development of CHEU need to be followed-up for extended periods of time in sufficiently large cohorts to build on the current knowledge and assist in developing intervention strategies to improve the morbidity and mortality of this vulnerable population. Research in diverse contexts and populations will be needed to create a comprehensive and representative picture of the long-term health prospects of CHEU.

## Data Availability

Not applicable.
